# Muscle modes of the equestrian rider at walk, rising trot and canter

**DOI:** 10.1371/journal.pone.0237727

**Published:** 2020-08-18

**Authors:** Marc Elmeua González, Nejc Šarabon

**Affiliations:** 1 Faculty of Health Sciences, University of Primorska, Koper, Slovenia; 2 S2P, Science to Practice, ltd., Laboratory for Motor Control and Motor Behaviour, Ljubljana, Slovenia; University of Belgrade, SERBIA

## Abstract

Equestrian sports have been a source of numerous studies throughout the past two decades, however, few scientists have focused on the biomechanical effects, including muscle activation, that the horse has on the rider. Because equitation is a sport of two (the horse-human dyad), we believe there is a need to fill in the knowledge gap in human biomechanics during riding. To investigate the differences between novice and advanced riders at a neuromuscular level we characterized the motor output of a set of riders’ key muscles during horse riding. Six recreational riders (24 ± 7 years) and nine professional riders (31 ± 5 years) from the Spanish Classical School of Riding (Lipica) volunteered to take part in this study. Riders’ upper body, core and lower limb muscles were monitored and synchronized with inertial data from the left horse’s leg at walk, rising trot and canter. We used principal component analysis to extract muscle modes. Three modes were identified in the advanced group whereas five modes were identified in the novice group. From the novice group, one mode united dorsal and ventral muscles of the body (reciprocal mode). Advanced riders showed higher core muscles engagement and better intermuscular coordination. We concluded that advanced horse riding is characterized by an ability to activate muscles contralaterally but not reciprocally (dorsal-ventral contraction). In addition, activating each muscle independently with different levels of activation, and the ability to quickly decrease overall muscle activity is distinctive of advanced riders.

## Introduction

Equestrian sports are a unique sort of activity during which two athletes (the horse-rider dyad) with different aspirations, physical and mental qualities work together to achieve success. Coordination within this dyad is an essential part of these sports, and it should be noted that the coordination must go in both directions (i.e. from the horse to the rider and vice versa). Equitation training regimens have traditionally focused on working with the horse at all times, either from the ground or mounted. In a similar way, in the past two decades science has been increasingly gaining interest to study the kinematics of horseback riding and its relation to performance and health risks of the horse associated to training. However, the literature has been focused on the effects that the rider has on the horse, yet only anecdotally has science gone the opposite direction when studying the dyad [1,2], leaving a big paucity of knowledge regarding rider biomechanics.

Kinematics of the horse’s trunk are gait-dependent [3,4]. In trot, the horse’s trunk ascends from mid-stance to the beginning of the flight phase and early stance, then the horse’s back descends during the second half of the flight phase and early stance. [1,2]. As a result, the simultaneous stance phases created by the diagonal steps (i.e. left hind leg—right foreleg, or right hind leg—left foreleg) provoke a pair of concurrent vertical movements of the cranial and caudal parts of the horse’s trunk [4]. Conversely, walk and canter gaits result in the horse's trunk rotating about the medial-lateral axis [4]. In order to achieve good horse-rider harmony and a stable seat, a good ability of the rider to coordinate with such motion kinematics is crucial [5–7]. A good stable seat, will ultimately lead to effective communication with the horse [8–10].

For the rider to achieve a movement that adapts to the horse’s motion, the central nervous system (CNS) needs to organize the numerous degrees of freedom (DOF) of the musculoskeletal system. That is, for a given task the CNS has many movement options and combinations that will lead to the exact same outcome [11]. For example, if we want to place our hand on a three-dimensional plane, we only need three axes to determine its position. However, the human arm has seven joints through which the CNS will determine the position of the hand. Hence, to place our hand at a given position within the three-dimensional plane, many arm configurations are available to the CNS that will lead to identical outcomes [12–14]. This is known as motor redundancy and was first introduced by Nikolai Bernstein in 1967 [15]. Many scientists have gained interest in this approach since the early 2000s [11,13,16–21]. The most common answer to how the CNS deals with these multiple anatomical, kinematic and neuromuscular redundant degrees of freedom (DOF), is that motor synergies serve as a mean for reducing such DOFs at the control level. Several studies have used correlation techniques and matrix factorization methods in an attempt to reduce the sets of variables that are involved in human motion through the course of action. The most common methods for the extraction of muscle synergies include principal component analysis (PCA) [22,23], non-negative matrix factorization [18] or individual component analysis [24].

To approach this complex interaction, we have opted to analyze the rider’s muscle modes (M-modes) with PCA, as it has been shown to be an effective means of obtaining a non-redundant set of variables from phenomena with high levels of dimensionality [25] and has been previously used in motor control studies that aimed to reduce the dimensionality of the motor redundancy problem [23,26,27]. Through this methodology we identified co-varied changes in muscle activations that could be sings of those muscles belonging to a mode during the time course of walking, trotting and cantering gait cycles in experienced and recreational riders. We have also included some basic surface electromyography (sEMG) analyses that will help understand how the human muscles work in order to face external perturbations to the body posture. Furthermore, understanding the human M-modes will also allow us to understand how riders can better harmonize with the horse’s motion, which will lead to improved balance and communication with the horse [8,9], decreased injury risk and, ultimately, higher performance [28].

The aim of this study is to describe the interaction and coordination of human muscles, namely M-modes, when exposed to equestrian movement perturbations elicited by the horse at walk, rising trot and canter. Moreover, this paper compares how M-modes of advanced riders differ when compared to novice riders in an attempt to better understand the training needs of the human side of the dyad. We hypothesized that advanced riders (i) have better adapted muscle activation patterns, (ii) higher core muscle activity and (iii) less antagonist coactivation.

## Materials and methods

### Participants

Six recreational riders (24 ± 7 years; training 2–4 hours/week), and nine professional riders (31 ± 5 years; training > 35 hours/week) from the Spanish Classical School of Riding of Lipica volunteered to take part in this study. All participants were informed of the procedure of the study and signed an informed consent waiver. The National Medical Ethics Committee (Ministry of Health, Ljubljana) gave full approval to the project according to the declaration of Helsinki (approval number: 0120-346/2018/3).

Four horses (15 ± 3 years), one show-jumping Anglo-Arabian (horse 1; level of competition CSN**) and three dressage Lipizzaners (horses 2, 3 and 4; Spanish Classical School) of similar morphological characteristics, performed the trials. All horses showed self-carriage capacities and calm, steady gaits. Approval from a veterinarian was given for each horse before it had taken part in the study.

### Experimental protocol

A cross-sectional, single-visit study design was used. Each rider completed the visit with horse 1 (recreational riders) and horse 2, 3 and 4 (professional riders). Every rider had previous experience with the allocated horse. All the measurements were performed in a horse arena and lasted < 1.5 h. After obtaining the informed consents and installing the EMG sensors, the riders were first asked to perform a set of 3 maximal voluntary contractions (MVC) for each muscle, for normalisation purposes. Each MVC was performed with manual resistance as described elsewhere [29]. After a standardized warm-up of the horse and rider, participants were asked to ride on a 30-m straight line, with minimal aids and aiming for self-carriage of the horse. Each rider had to repeat this procedure until 60 clean horse-strides were recorded at walk, trot and canter. To prevent any potential injury of the horse, the three gait conditions were not randomized and were performed in this order. Riders were asked to perform rising trot and collected canter, always on the right lead. If the horse was disunited or performed leg changes, the whole 30-m repetition was eliminated. Upon completion, cool down of the horse with loose reigns at walk was performed.

### Horse set-up

An all-purpose saddle (Zaldi Olympic, Spain) was used for all horses. Saddle fit was checked according to the standardized agreements proposed elsewhere [30], with a 0º seat angle. Stirrup length variability was kept to a minimum by asking the riders to match their knee with the knee roll and keep an angle as close as possible to 90º at the knee joint. Each horse was ridden with their own bridle, which in all cases was mounted with a simple snaffle bit. Noseband pressure was checked with a commercially available noseband pressure gauge.

Closed, neoprene horse boots were installed on the front legs. The boot on the right front leg was instrumented with an inertial measuring unit and the boot of the left front leg was taped in the same way in order for the horse to feel the same amount of compression in both legs, to avoid imbalances on the gait.

### Surface electromyography (sEMG)

sEMG activity of right (R) and left (L) erector spinae (ES), multifidus (MF), abdominal external oblique (EO), vastus lateralis (VL), biceps femoris (BF) and gastrocnemious lateralis (GL) muscles were recorded with a wireless sEMG recording system (Delsys Tringo, Delsys, USA). The electrodes, attached with a self-adhesive interface and impregnated with conductive cream, were placed longitudinally and aligned to the underlying muscle fibre arrangement and were located according to the recommendations of Surface EMG for Non-Invasive Assessment of Muscles [31] and Vera et. al [29]. Skin was previously shaved, abraded and cleaned with alcohol to minimize impedance (Frère and Hug, 2012) (< 5 kΩ). The Raw sEMG signals were pre-amplified (gain 300) with a built-in amplifier and recorded at a sampling rate of 1100 Hz.

### Data analysis

All signals were processed *ex situ* using EMGWorks 4. (Natick, USA). EMG signals were rectified and bandpass filtered with a 10/500 Hz [32], second-order, zero-lag Chebyshev filter [33]. EMG amplitude was obtained from an RMS linear envelope (.02-sec window; .01-sec window overlap) and normalized to the peak EMG recorded during the corresponding MVC. In order to avoid noise interference of EMG signals on the shape of the principal components, we excluded EMG recordings with mean values below 2 μV [19].

All the signals corresponding to non-discarded horse strides were concatenated, time normalized (0 to 100 arbitrary units) and averaged to obtain a representative cycle for each rider at each gait that was comparable both across subjects and gaits. For the selection of the starting and ending point on the determination of one full horse stride, IMU data from the right forelimb of the horse was filtered with a built-in impact filter (EMGWorks 4, Natick, USA). One stride was defined as the time between two successive ground contacts of the right front limb, as described elsewhere [4]. Briefly, signals were visually inspected, and a threshold value was set at the lowest recorded acceleration that clearly corresponded to horse-leg impacts. This value changed across riders and gaits due to varying motion kinematics.

Bursts of activity were assessed with a custom-written MATLAB 9.4.0 (Natick, USA) code. Threshold values of 25% of the maximal MVC amplitude for each muscle and subject were used to determine individual onsets and offsets of muscle activity [34]. The active phase (AP) has been defined as the duration between the onset and the offset of the muscle activity. Signal was additionally analysed using the following parameters: maximal amplitude of the linear envelope (Ûmax), position of the gait cycle where Ûmax was achieved (PCYC), average amplitude of the 60 computed strides (Ûmean) and Ûmean of the active phase (Ûmean_AP_). A ratio of riders found on AP in each part of the cycle for each muscle and group has been calculated by dividing the number of riders that were activating each muscle above 25% of MVC amplitude by the total amount of riders of their corresponding group.

### Extraction of principal components

For each gait and subject, PCA was performed using IBM Corp. SPSS 23 (Armonk, USA) in order to obtain M-modes as described by Latash [11]. Outliers were removed from the test. Extraction was based on Eigenvalues greater than 1, with a varimax rotation (25 maximum iterations). Barlett test’s hypothesis was rejected and therefore demonstrated the existence of latent factors in the dataset. Kaiser-Meyer Olsen (KMO) revealed an adequate sample size for PCA (> 0.6). Muscles with factor loadings > .55 were considered as contributors for specific factors [35]. Components were examined and, unless they explained >3% of the total variance, they were dropped [19]. M-modes interpretation was put in the context of postural control around the dorsal/ventral sides of the ankle, knee and hip joints as described by Asaka et al. [36]. Reciprocal modes were characterized by a parallel scaling of dorsal or ventral muscle activity levels across several joints [36] whereas co-contraction modes corresponded to parallel changes in the activation levels of muscles with opposing actions [26]. Mixed modes involve co-activation on a joint and two more muscles significantly loaded [36].

### Statistical analysis

All statistical analyses were performed with IBM Corp. SPSS 23 (Armonk, USA) and MS Excel 16.24 (Redmond, USA). EMG data was analyzed with a three-way ANOVA (group × gait × muscle). Further analysis by means of 2-sample, 1-tailed t-tests were conducted to observe significant differences between groups and muscles. The Shapiro-Wilk test of normality revealed a non-normal distribution of Ûmean and Ûmax. A logarithmic transformation was applied to both variables. Values are reported as mean ± SD.

## Results

### Electromyography

**Fig 1:**
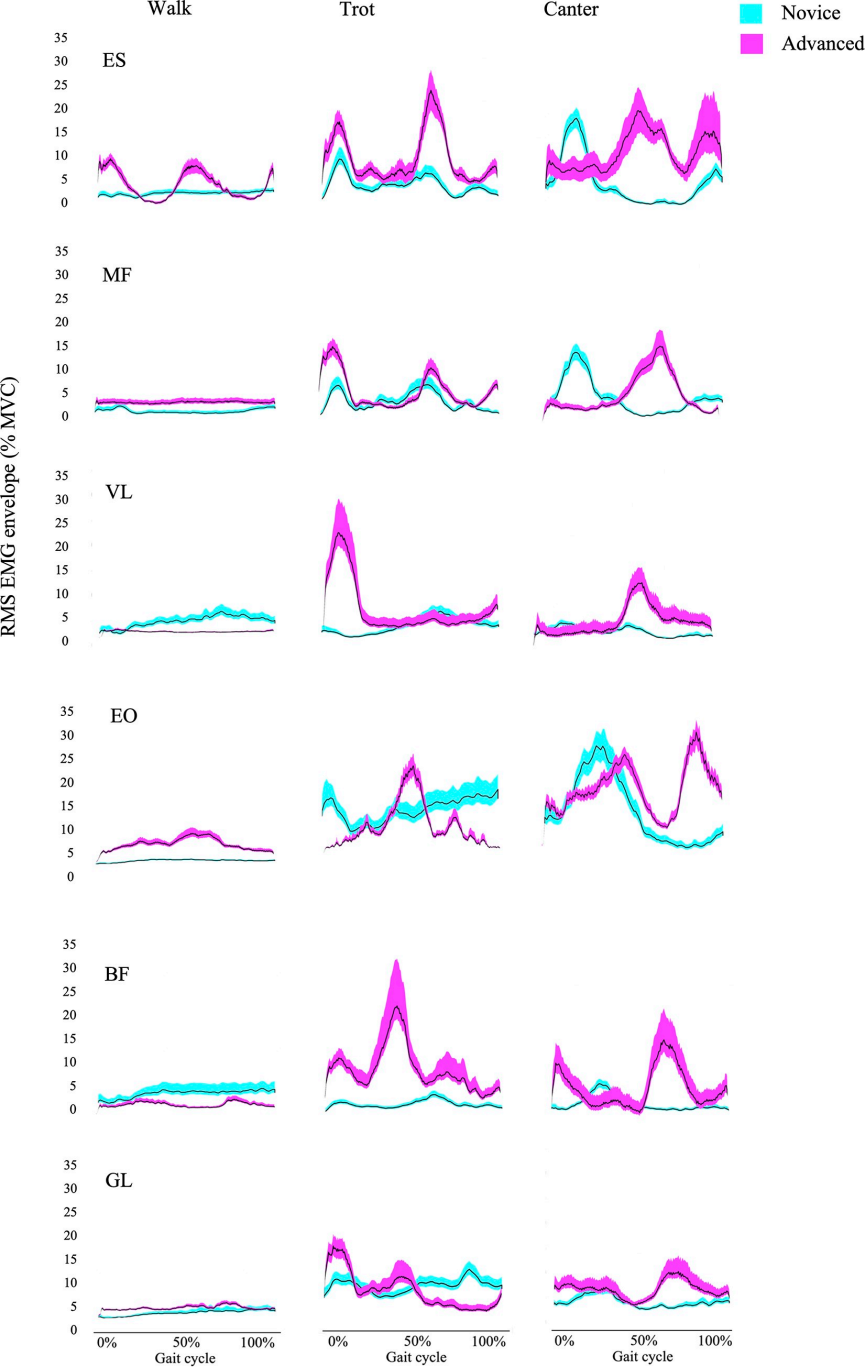
Representative data of mean +/- SD EMG amplitude of the left muscles of 60 averaged cycles at walk, trot and canter.

Representative data of mean +/- SD EMG amplitude of the left muscles during an entire horse stride at walk, trot and canter are presented in Fig 1. Signal to noise ratios (SNR) given by the formula 20 log (Power of signal*Power of noise-1) are presented in Table 1.

**Table 1:** Signal to noise ratio (SNR) of EMG signal recorded during the three MVCs and the experimental trial series, for each muscle.

	MVC SNR			Series SNR		
LES	1.32	±	.33	1.36	±	.18
RES	1.45	±	.44	1.31	±	.22
LMF	1.23	±	.39	1.23	±	.34
RMF	1.27	±	.12	1.17	±	.17
LGL	1.28	±	.25	1.12	±	.26
RGL	1.25	±	.23	1.08	±	.24
LEO	1.29	±	.14	1.22	±	.02
REO	1.29	±	.14	1.22	±	.02
LVL	1.29	±	.14	1.22	±	.02
RVL	1.29	±	.14	1.22	±	.02
LBF	1.29	±	.14	1.22	±	.02
RBF	1.29	±	.14	1.22	±	.02

**Fig 2:**
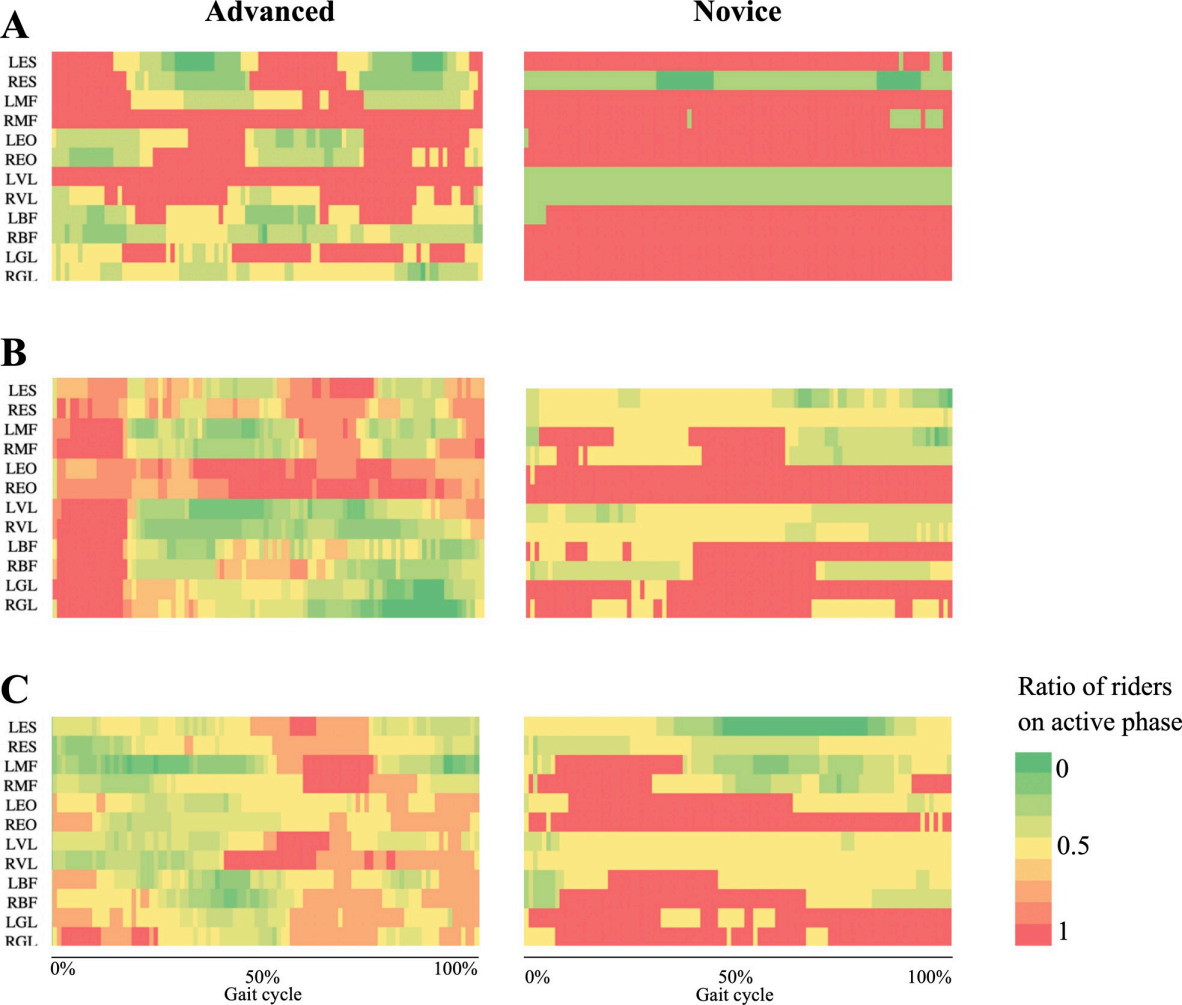
Heatmap of the muscles found on the active phase of EMG signal during one averaged horse stride expressed as a ratio of riders that were in the active phase. Heatmaps correspond to (A) walk, (B) trot and (C) canter.

Graphical representation of the percentage of riders mean AP of independent muscles throughout the cycle are shown in Fig 2 and visually revealed a more persistent, thus inefficient, pattern of muscle activity in novice riders compared to advanced riders.

A three-way ANOVA was conducted that examined the effect of proficiency level, gait and muscle on Ûmean, Ûmax and Ûmean_AP_. On all variables statistically significant differences between groups, gaits and muscles were observed, F (1, 418) = >10.53, p < .00; F (2, 418) > 31.99, p < .00; F (11, 418) > 2.23, p < .01, respectively. Summary descriptive data for overall Ûmean, Ûmax and ÛmeanAP across riders and muscles is presented in Table 2.

**Table 2:** Summary descriptives of overall EMG amplitude.

group	gait	Ûmax	Ûmean	Ûmean_*AP*_
advanced	canter	58.47 ± 26.96	7.28 ± 6.78	9.80 ± 8.99
	trot	34.75 ± 26.91	8.15 ± 5.64	9.61 ± 7.24
	walk	55.21 ± 29.81	3.23 ± 19.02	4.72 ± 8.67
novice	canter	34.08 ± 21.24	6.32 ± 4.07	7.50 ± 6.37
	trot	50.50 ± 28.89	5.51 ± 3.34	6.13 ± 3.75
	walk	53.42 ± 29.61	2.92 ± 1.74	2.99 ± 1.77

All values are expressed as mean ± SD in %MVC.

T-tests revealed that, (a) at walk, advanced riders showed a significantly higher Ûmax of RES and LEO (p < .03) and Ûmean_AP_ of LEO (p < .02) compared to novice riders, and LES PCYC occurred significantly earlier than RES PCYC in advanced riders but not in novice riders; (b) at trot, Ûmax of LVL and RVL was significantly higher in advanced riders compared to novice riders (p < .02), and PCYC of LVL, RVL, LBF, RBF, LGL and RGL occurred significantly earlier in advanced riders than novice riders (p < .03), however, LES and RES PCYC occurred later in advanced riders (p < .00). Advanced riders achieved a higher Ûmean_AP_ on LES, LVL and LBF compared to novice riders (p < .05) but had lower RVL (p < .02); (c.) at canter, advanced riders had higher Ûmax of the RBF (p < .00) compared to novice riders, and PCYC of LES, RES, LMF, RMF, LVL and RVL occurred later in advanced riders compared to novice riders (p < .00). A summary of t-tests results can be found in Table 3.

**Table 3:** Summary of *t*-tests results.

	Ûmean_*AP*_		Ûmax		PCYC	
	Advanced	Novice	Advanced	Novice	Advanced	Novice
Walk	LEO		LES, REO		LES occurred earlier than RES	
Trot	LES, LVL, LBF	RVL	LVL, RVL		LVL, RVL, LBF, RBF, LGL, RGL	LES, RES
Canter			RBF			LES, RES, LMF, RMF, LVL, RVL

Displayed muscles are those that achieved a significantly higher mean EMG amplitude during the active phase (Ûmean_AP_), higher maximal EMG amplitude (Ûmax) and earlier position of the gait cycle where Ûmax was achieved (PCYC) (p < .05).

### Muscle modes

**Fig 3:**
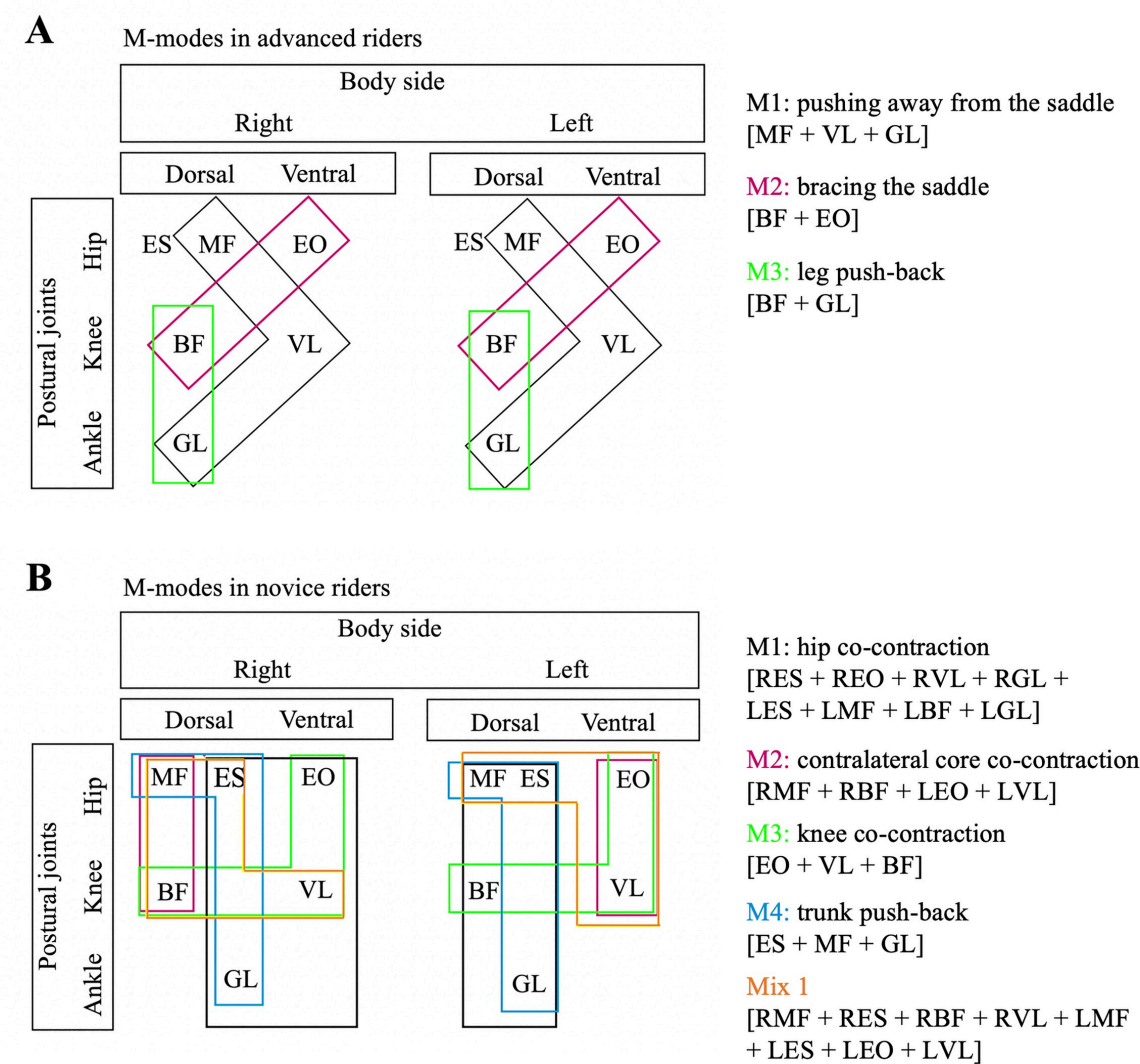
Diagram of the classification of muscle modes (M-modes) based on PCA results. Four reciprocal modes were identified in the advanced group (A). One reciprocal mode, three co-contraction modes and one mixed mode (B) were identified in the novice group.

PCA reduced the dimension of advanced riders’ M-modes to 2 factors (m-modes) at walk and 3 at trot and canter. On novice riders, PCA led to 2 factors at walk, 3 at trot and 2 at canter. Table 4 shows the outcome results of the PCA and factor loadings for each muscle and M-mode. Throughout the whole three trials (i.e. walk, trot and canter) three reciprocal M-modes were identified in the group of advanced riders (Fig 3A). One reciprocal mode, three co-contraction modes and one mixed mode were identified in the novice group (Fig 3B)

**Table 4:** M-modes of advanced and novice riders at walk, trot and canter with factor loadings > .55 corresponding to each muscle and M-mode.

	Walk					Trot					Canter				
	Advanced		Novice		Advanced			Novice			Advanced			Novice	
*M*-*mode*	1	2	1	2	1	2	3	1	2	3	1	2	3	1	2
**LES**	.52	-.80	.69*		.84			.90			.71		.59*		.97
**RES**	.59	-.69	.69		.77			.97*			.97				.93
**LMF**	.86			.80	.94			.68	.69*		.93				.91
**RMF**	.88		.79		.94					.88	.87				.96
**LEO**		.87	.87*				.94		.94*				.89*	.94	
**REO**		.80		.89			.83	.80*					.90	.90	
**LVL**	.63		.86		.73			.72*		.58	.85*			.91*	
**RVL**	.89			.90	.73			.89			.58		.59	.91*	
**LBF**		.79		.79		.86		.89*			.56*	.75		.83*	
**RBF**		.74	.80			.81			.83			.83		.91*	
**LGL**	.64		.80			.88				.88		.92			.94
**RGL**	.73		.77			.75			.96			.80			.64

*Muscles in co-activation with their antagonist within a component.

## Discussion

The purpose of this study was to create a muscle activation profile of the horse rider at walk, trot and canter and compare professional to novice riders. We hypothesized that advanced riders would have (i) better adapted muscle activation patterns, (ii) higher core muscle activity and (iii) lesser antagonist coactivation. Basic EMG analysis has supported our first and second hypotheses while PCA analysis supported our third hypothesis.

At first glance, results point out a trend for higher and earlier activation of core muscles by the advanced riders at walk. These can potentially demonstrate that work during walk is an undervalued asset amongst amateurs, and that full engagement of core muscles at all times during a riding session is characteristic of advanced riders. This supports the findings by Terada et al. [1] which point out the importance of abdominal muscles while riding, and suggests that core training can be a beneficial implementation to any riding discipline. During trot, the same pattern was repeated. This time, however, lower limb muscles were more important, most particularly in the advanced group. Stirrups are a means of supporting the rider’s legs, not the weight of the rider [37] however advanced riders achieved higher VL activity suggesting a stronger use of the stirrups at trot when compared to novice riders. It has previously been reported that, at rising trot, advanced riders maintain a more straight and vertical trunk whereas novice riders lean forward ahead from the vertical line [4,37,38]. Consequently, one explanation to such differences in VL activity could be that, as advanced riders sustain their weight on the stirrup through a more perpendicular vector, they must as well generate higher force levels. Conversely, van Beek et al. [39] reported that the height of the force peaks measured at the stirrup was not influenced by riding experience or level. Therefore, increased VL activity could be linked to leg position rather than higher force production. Hip rotation and its relation to EMG activity of the lower limb muscles is a controversial source of research with most studies pointing to increased VL activity with external rotation of the hip [40–42]. A recent study by Babadi et al. [43] suggests that VL achieves a higher activation in both internal and external rotations of the hip and also points out the influence of hip hinge and knee position on VL activity. Therefore, differences in VL activation could also be explained by wider hip angles of the advanced riders compared to those observed in novice riders [37,44,45] and greater internal rotation of the knee [46]

Throughout the cycle, advanced riders showed a later maximal activation of the upper body muscles, which could be explained again by a more vertical trunk alignment [4,37,38] which prevents the activation of trunk stabilizers involved in voluntary body sways [23,26] that would be expected in novice riders when leaning forward. During canter, maximal amplitude of back and leg muscles are attained later in the cycle in advanced compared to novice riders, suggesting a similar pattern of balance. This points out that advanced riders have a higher ability to anticipate the impact at a neuromuscular level when compared to novice riders and is consistent with the findings by Münz et al. [4] who reported that advanced riders reached their maximal dorsal pelvis tilt later than novice riders when cantering.

Fig 2 provides a visual representation of the muscles being activated in each part of the gait cycle. It is worth noting that walk and trot are both gaits where the advanced riders differed notably from the novice riders. At walk, we can clearly observe in the advanced group a pattern that alternates activation on the trunk flexors (EO) and trunk extensors (MF, ES). In turn, the novice group shows a constantly activated pattern of muscle activity. Münz et al. [4] found that the main difference of pelvic trajectories between advanced and novice riders at walk was a more congruent anterior-posterior rotation trajectory production by the advanced riders, which could be explained by these findings. We believe that the muscle activation pattern shown by the novice group allows the riders to stabilize their COG yet it also constricts the horse’s motion. It is important to acknowledge this finding because any skill to be taught to the horse will always need to go through walk at some point in the horse’s learning process. It is therefore of great interest that a rider can alleviate the big mental and physical load of teaching new skills to a horse by being less restrictive in the movement.

At trot, we found a similar pattern. Advanced riders show a more defined and relaxed pattern of muscle activation. In this gait, leg muscles (BF, VL, GL) are activated at the beginning of the stride -which corresponds to the right front leg touching the ground- as the rider lifts the pelvis. In a similar way to the walk muscle patterns, back extensors (MF, ES) and flexors (EO) of the advanced riders alternate in the active phase, this time in a more progressive fashion. Terada et al. [1] suggested that the external obliques might work in conjunction with the rectus abdominis, which increases intra-abdominal pressure [47,48], utilizing this rise in pressure as a mechanism for stabilizing the trunk [49]. Novice riders showed a similar pattern, however, there was again more activity in all muscles at all times, and less leg muscles activity at the beginning of the cycle (i.e. when the horse’s right front leg hits the ground).

At canter, novice riders activated ankle extensors (GL), followed by outside/left leg extensor (LVL) and inside/right back flexor (REO), and finally they activate left lower back and right upper back extensors (LMF, RES). These outcomes support previous work that described the canter as a gait that elicits a single cycle of flexion-extension of the thoracolumbar spine [50] and points out the existence of an additional mediolateral bending cycle. In contrast, a higher knee flexor (BF) activity can be observed in the novice group, which could be explained by a lack of independent seat and excessive leg gripping. Moreover, the back muscles are more active throughout the entire gait cycle.

With regards to the PCA results: during trot, the novice riders shared a higher number of muscles across factors (LMF, LVL) compared to advanced riders (none), while the latter shared three muscles (LES, RVL, LBF) during canter and two muscles (LES, RES) during walk, compared to no sharing observed in the novice group either at walk or canter. We expected advanced riders to behave in a less redundant manner (i.e. less shared muscles across factors). It is worth noting that novice riders show a more chaotic muscle distribution across modes, while advanced riders show an ability to synergistically activate both sides of the body at the same time. In other words, advanced riders’ M-modes include both right and left versions of the same muscles, while novice riders might not have the ability to split motor neural output contralaterally and end up using only one side of the body at a time. These results, combined with the basic sEMG analysis, demonstrate that advanced riders have the ability to activate muscles contralaterally at different magnitudes, while novice riders follow an “on/off” model of muscle activation in which they either activate one side or the other, which can compromise the general tone of the body. It is worth noting that advanced riders did not show any co-contraction nor mixed mode, while novice riders did only show one reciprocal mode (Fig 3). Such finding could be demonstrating that co-contraction is one limiting factor to horse riding expertese. Previous studies on co-contraction M-modes showed that muscle co-contraction patterns are predominant in postural tasks performed under challenging conditions [51] and unpredictable postural perturbations [52] which can explain why less experienced riders show higher levels of co-contraction, as they might have a lower ability to predict and adapt to the movement of the horse.

These findings bring an interesting perspective to motor learning of the equestrian rider. In 2011, Trupin et al. [53] compared muscle synergies during rowing of trained and naïf rowers. They found no difference in the organization of muscle activation patterns. From their results they hypothesized that expertise in rowing might not be directly related to the shape and timing of muscle synergies. Conversely, Asaka et al. [36] tested the hypothesis that activation patterns could be improved through practice and found that after five days of practice of load-release tasks on an unstable board, subjects were able to better maintain balance and reduce the co-contraction M-modes. They concluded that M-modes are a flexible set of elemental variables, which the CNS uses to ensure COP location. Practice can influence both the pattern and the composition of M-modes, which results in stronger COP-stabilizing synergies. These findings point out to the idea that M-modes are more flexible when dealing with balance-related tasks, and therefore equitation could be an activity that can potentially generate M-mode adaptation.

## Conclusion

The biomechanical relationship of the horse with the rider is fairly unknown when it comes to the effects of the horse on the rider. Knowledge on what makes a good rider is based on anecdotal evidence and it varies largely across countries, riding centers and even riders. To the best of our knowledge, this study is the first to investigate how experienced riders differ from amateurs at a neuromuscular adaptation level and represents an important starting point to understanding how the human body works during horse-back riding at walk, rising trot and canter. The primary conclusions that can be drawn from this set of data are that (a) professional riders have higher overall muscle tone and (b) are using their core muscles to a greater extent than novice riders. Furthermore, (c) novice riders do not have the ability to activate muscles contralaterally and independently with different magnitudes, making them less adaptive to the movements of the horse and, most probably due to lack of self-body awareness, (d) they do not show the capability to move their own COG around the saddle to come in and out of alignment with the horse’s COG when desired.

These four conclusions will be of great importance to any equestrian coach or strength and conditioning coach working with horse riders, as it points out four physical qualities that can be improved through training off the horse and could potentially increase performance of the dyad without increasing training load of the horse, thus preventing overtraining and/or injury of the animal. Further research on the benefit of off-horse training of these physical qualities would be beneficial for the equitation community.

As it has been previously demonstrated, the implementation of a core training program can have a significant impact on riding performance [54]. These researchers found that performing three weekly 20-minute sessions of a sport-specific training regime was enough to elicit substantial adaptations in the rider over the course of eight weeks. In addition to Hampson and Randle’s work, the present study suggests that an equitation-specific training regime should include:

- Whole body muscular toning exercises with special attention to core stability
- Bilateral dissociation exercises of both upper and lower extremities
- COG awareness exercises

## Limitations

It should be acknowledged that this study has potential limitations. The main drawback is the use of surface electromyography and its associated limitations discussed elsewhere [55,56] such as low signal-to-noise ratios seen in the multifidus and gastrocnemius lateralis muscles (Table 1). However, the election of this method responds to the need of a non-invasive method, near to impossible to feel by the subject, that provides us with enough accuracy and repeatability for the purpose of this study. The second limitation we face in any study involving a ridden horse is the unpredictable and uncontrolled framework due to the living animal. We tried to mitigate such limitation by electing calm and experienced horses, yet we will never be able to have absolute control over such environment. Finally, the use of several horses for the study, which is a necessary step into preserving animal welfare, can potentially influence the results of each rider. This is a limitation that we will always encounter in equitation science as no rider wants a horse to perform such exercises more than once or twice per day nor for extended periods of time (i.e. weeks or months), as this could influence both mental and physical performance of the horse. Finally, all novice riders rode a single horse with showjumping background whereas the advanced group rode three Lipizaner horses, which can potentially lead to more variability in the advanced group compared to the novice group.

## Supporting information

S1 Data (XLSX)
